# Kaniadakis Entropy Leads to Particle–Hole Symmetric Distribution

**DOI:** 10.3390/e24091217

**Published:** 2022-08-30

**Authors:** Tamás S. Biró

**Affiliations:** 1 Wigner Research Cenrer for Physics, H-1121 Budapest, Hungary; biro.tamas@wigner.hu; Tel.: +36-20-435-1283; 2 Hungarian Institute of Physics, University Babeş-Bolyai, RO-400084 Cluj, Romania; 3 Complexity Science Hub, A-1080 Vienna, Austria

**Keywords:** Kaniadakis entropy, kappa statistics, Tsallis distribution, KMS relation

## Abstract

We discuss generalized exponentials, whose inverse functions are at the core of generalized entropy formulas, with respect to particle–hole (KMS) symmetry. The latter is fundamental in field theory; so, possible statistical generalizations of the Boltzmann formula-based thermal field theory have to take this property into account. We demonstrate that Kaniadakis’ approach is KMS ready and discuss possible further generalizations.

## 1. Introduction

Remembering when statistical physics passed the “classical” Boltzmann–Gibbs distribution view of exponential dependence on individual energies, one ought to formulate a few general statements. Certainly, a generalization [1,2], in addition to including the original classical formulas in some limits, can be infinite. In physics, however, nature gives us several clues as to which generalization is more useful, moving beyond a pure mathematical construction.

Generalizations of entropy formulas replace the logarithm with another function, and the change from an exponential function in the equilibrium or in other way stationary distributions to something else are the inverse operation to this. Informatics studies were pioneering in generalizing the entropy formula of Boltzmann in the 1950s and 1960s [3,4], while the thermodynamical consequences have been more vividly studied since the 1980s [5,6,7]. Here the power-law tailed distribution, originally considered an approximation to the exponential by Euler and in particle physics by Hagedorn as a “cut power-law”, in the beginning did not have any physics rationale aside from its aimed application.

Another widespread nonexponential distribution, also extrapolating to power-law tails is given in the kappa statistics initiated by Kaniadakis. It is motivated by relativistic kinetics in plasmas, and its most renowned applications are also related to plasmas. While it can be mapped to an exponential of the rapidity, replacing the energy variable by a rapidity-like one as the argument, its high energy tail seems to show remarkable success in application to real world data. A general presentation of kappa statistics basics can be read in [8]. The relation to special relativity is discussed in [9], and to the Boltzmann equation in [10]. Fractional statistics in kappa statistics are dealt with in [11], nonlinear kinetics in [12], and a general review about the physical origins in [13].

In this paper we point out that based on a particular property of the mathematical formula appearing in kappa statistics, this form is able to reflect particle–hole symmetry, an important ingredient in field theory and particle physics. The underlying concept in field theory namely assumes a symmetry between particles and antiparticles, called CPT symmetry, changing charges, parity, and time direction to its opposites. The physical laws should not change in an antimatter world relative to the original one. The mathematical formulation of the time-dependent expectation values for elements of statistical ensembles in field theory is related to the use of a statistical operator. Whenever the exponential function of energy is generalized in the statistics, the corresponding statistical operator is also no more the Boltzmann–Gibbs exponential of the Hamiltonian operator. Still, the CPT reflection should not change the physical conclusions. Therefore, it is essential that the generalization of the exponential function shows similar reflection properties to the original Euler number-based function.

## 2. Kaniadakis’ Generalized Exponential

There are several generailzed entropy formulas and corresponding canonical distributions [1,2,3,4,5,6,7,8,9,10,11,12,13,14]. At their core, they can be viewed as the generalization of the logarithm and exponential functions while keeping their inverse roles. However, the inverse relation between the exponential of *x* and −x is, in general, lost.

The Boltzmann–Gibbs energy distribution at a fixed temperature utilizes the Euler exponential function, which has the property, exp(−x)=1/exp(x). Accordingly, its inverse, the logarithmic function, satisfying both ln(exp(x))=x and exp(ln(x))=x, also satisfies

(1) $$\ln\frac{1}{x}\;=\;-\ln x.$$

This is important in the use of the Boltzmannian entropy formula [15],

(2) $$S/k_{B}\;=\;\sum_{i}p_{i}\ln\frac{1}{p_{i}}\;=\;-\langle \ln p_{i}\rangle$$

with the probability $p_{i}$ of being in the *i*-th state, a real number between and including zero and one. The above formula is valid only if the probability set is normalized, i.e.,

(3) $$\sum_{i}p_{i}\;=\;1.$$

Otherwise, the leading order terms while applying the Stirling formula [16,17,18,19] to the permutation entropy [20,21,22,23] would not cancel. These basic features of this construction lead to an overall nonnegative entropy and to its concavity property [24,25,26,27,28].

When generalizing, such as in some axiomatic approaches, the properties have to be saved, while much less attention is paid to the ln(1/x)=−lnx relation. In fact, some of the suggested extensions to the exponential and logarithm function satisfy such a relation, others do not. Let us review a few of them.

The Kaniadakis’ κ-exponential [8],

(4) $$e_{\kappa }\left(x\right)\;=\;{\left(\kappa x+\sqrt{1+\kappa^{2}x^{2}}\right)}^{1/\kappa },$$

satisfies the relation

(5) $$e_{\kappa }\left(-x\right)\;=\;1/e_{\kappa }\left(x\right).$$

On the other hand, the Tsallis *q*-exponential [5], designed to have a power-law tail relying on Euler’s approximating formula for the exponential for n=1/(q−1),

(6) $$e_{q}\left(x\right)\;=\;{\left(1+(q-1)x\right)}^{\frac{1}{q-1}},$$

behaves differently when reflecting the argument:

(7) $$e_{q}\left(-x\right)\;=\;\frac{1}{e_{2-q}\left(x\right)}\;\neq \;\frac{1}{e_{q}\left(x\right)}.$$

Here $\lim_{\kappa \to 0}e_{\kappa }\left(x\right)=e^{x}$ and $\lim_{q\to 1}e_{q}\left(x\right)=e^{x}$ are the limits leading back to the traditional exponential.

It is easy to construct another class of functions based on a symmetric use of the Tsallis exponentials, which satisfies the product formula searched for in [29].

(8) $${\tilde{e}}_{q}\left(x\right)\;=\;\frac{e_{q}\left(x/2\right)}{e_{q}\left(-x/2\right)}$$

namely delivers

(9) $${\tilde{e}}_{q}\left(-x\right)\;=\;\frac{e_{q}\left(-x/2\right)}{e_{q}\left(x/2\right)}\;=\;\frac{1}{{\tilde{e}}_{q}\left(x\right)}.$$

This works only with the halved argument ratio definition.

The above sketched relation between the Kaniadakis’ exponential and a symmetric ratio of Tsallis exponentials can be generalized. We construct a *k*-exponential class based on a general function, $f_{k}\left(x\right)=a_{k}\left(x\right)+kxb_{k}\left(x\right)$, with both $a_{k}\left(x\right)$ and $b_{k}\left(x\right)$ being even functions of *x*. Then,

(10) $$e_{k}\left(x\right)\;=\;f_{k}{\left(x\right)}^{1/k}\;=\;{\left(a_{k}\left(x\right)+kxb_{k}\left(x\right)\right)}^{1/k}$$

with its reflected pendant

(11) $$e_{k}\left(-x\right)\;=\;{\left(a_{k}\left(-x\right)-kxb_{k}\left(-x\right)\right)}^{1/k}\;=\;{\left(a_{k}\left(x\right)-kxb_{k}\left(x\right)\right)}^{1/k}$$

satisfies

(12) $$e_{k}\left(x\right)\cdot e_{k}\left(-x\right)\;=\;1$$

only if

(13) $$a_{k}^{2}\left(x\right)\;=\;1+k^{2}x^{2}b_{k}^{2}\left(x\right).$$

Furthermore, having the traditional exponential in the k→0 limit, both $a_{0}$ and $b_{0}$ have to converge to unity. This leads to the following class of Kaniadakis type of deformed exponentials:

(14) $$e_{k}\left(x\right)\;=\;{\left(\sqrt{1+k^{2}x^{2}b_{k}^{2}\left(x\right)}+kxb_{k}\left(x\right)\right)}^{1/k}.$$

For a nontrivial $b_{k}\left(x\right)$ even function, we may consider an example:

(15) $$b_{k}\left(x\right)\;=\;\frac{1}{\sqrt{1-k^{2}x^{2}}}.$$

In this case, one obtains

(16) $$e_{k}\left(x\right)\;=\;{\left(\sqrt{\frac{1+kx}{1-kx}}\right)}^{1/k}\;=\;\frac{e_{q}\left(x/2\right)}{e_{q}\left(-x/2\right)},$$

with a power-law tail for an expression relating to the relativistic Doppler factor. On the other hand, this is equal to a symmetrized ratio at the half argument of Tsallis type deformed exponentials exactly with q=2k+1. In this interpretation, the $b_{k}\left(x\right)$ function is the Lorentz factor, with kx=v/c=tanhη being a velocity in units of the light speed. At the same time, $e_{k}\left(x\right)=e^{\eta /k}$. Hence, the rapidity variable η is additive due to the product of traditional exponential functions, and therefore, this delivers the mapping to the logarithm of the formal group: to the additive quantity belonging to the nonadditive rules generated by the deformed exponentials [22].

This additive variable, η, can also be constructed in the general case. Setting $kxb_{k}\left(x\right)=\sinh\eta$ and kx=g(η) as a general function, one has

(17) $$b_{k}\left(x\right)\;=\;\frac{\sinh\eta }{g(\eta )}$$

leading to $e_{k}\left(x\right)=e^{\eta /k}$. In our previous example, we had g(η)=tanhη.

After reviewing examples and generalization paths, we turn to the question of why it is so important to have the property $e_{k}\left(x\right)\cdot e_{k}\left(-x\right)=1$ in high energy physics and field theory in the next section. Further applications of Kaniadakis’ exponential [30,31,32] and a general approach to group entropy [33] provide the reader with further information on generalizing the exponential function and its use in data processing and interpretation.

## 3. Particle–Hole Symmetry

The Kubo–Martin–Schwinger (KMS) relation is central in thermal field theory [34,35,36]. Physically, it reflects the reinterpretation of negative energy states of a quantum particle as the corresponding positive energy state of an antiparticle. A hole in the negative energy continuum is a positive energy propagating particle with opposite momentum and charges.

In this paper, we briefly review a somewhat generalized version of the KMS relation, in order to make it clear that its validity extends beyond thermal equilibrium. This presentation is based on Ref. [37].

Quantum packages of energy and charge do propagate according to field theory as solutions to the field equation Green functions, i.e., propagators. Such propagators have a few subtypes according to retarded and advanced options in their causality structure, reflected in pole positions on the complex energy plane. Since an interacting particle in a finite time can never have an energy which would exactly follow from the solution of the classical free field equation, quoted as the dispersion relation between the frequency and wave number vector, the off-mass-shell behavior is a mirror of its quantum nature. This deviation from the special relativistic energy–momentum formula for a free point particle is well comprised in the spectral function.

Spectral functions can be defined and investigated generally among two quantum field operators, say $\hat{A}$ and $\hat{B}$, in the presence of a statistical operator, $\hat{\rho }$, by a time-Fourier transform,

(18) $$S_{AB}\left(\omega \right)\;=\;\int \;dt\;e^{-i\omega t}\mathrm{Tr}\left(\hat{\rho }\left[\hat{A}\left(t\right),\hat{B}\left(0\right)\right]\right).$$

The operators are taken in a time distant *t* from each other, utilizing the Heisenberg picture in field theory. The above definition tacitly assumes that the statistical operator, the statistical weight of states related to the Hamiltonian, is stationary. Whenever it contains a temperature parameter, such as β=1/T, or further parameters, such as κ or *q*, the spectral function will be also parametrized by them.

In a stationary state including but not restricted to thermal equilibrium, the time reversal and energy reversal properties of the AB-generalized spectral function should be studied. Indeed, in the definition Equation (18) the time-shift invariance is also assumed, which is equivalent to the conservation of the total energy. Meanwhile, the operators $\hat{A}$ and $\hat{B}$ can be evaluated on the observed subsystem, whose spectral function we consider.

When the operators, correlated by the selected spectral function, are also time-shift invariant and Hermitean, then the following symmetry properties are ensured:

(19) $$S_{AB}^{*}\left(\omega \right)\;=\;S_{B^{†}A^{†}}\left(\omega \right),\;S_{AB}\left(-\omega \right)\;=\;-S_{BA}\left(\omega \right).$$

Both properties utilize the time-shift invariance of the trace,

(20) $$\mathrm{Tr}\left(\hat{\rho }\hat{A}\left(t\right)\hat{B}\left(0\right)\right)\;=\;\mathrm{Tr}\left(\hat{\rho }\hat{A}\left(0\right)\hat{B}\left(-t\right)\right).$$

The Wigner transform of the [A,B]=AB−BA commutator’s statistical expectation value in a given quantum state shows similar properties. The Wigner function definition

(21) $$S_{AB}\left(x,p\right)\;=\;\int \;dq\;e^{\frac{i}{\hbar }p\cdot q}\;\langle \left[\hat{A}\left(x-q/2\right),\hat{B}\left(x+q/2\right)\right]\rangle ,$$

extends the above concept from a simple time coordinate to the spacetime coordinates *x* and corresponding four-momenta *p*. The dot in the exponent denotes the Minkowski scalar product, $p\cdot q=Eq_{0}-\vec{p}\cdot \vec{q}$. Now, in the 8-dimensional phase space, one has the properties:

(22) $$S_{AB}^{*}\left(x,p\right)\;=\;S_{B^{†}A^{†}}\left(x,p\right),\;S_{AB}\left(x,-p\right)\;=\;-S_{BA}\left(x,p\right).$$

Analogous to the Wigner (spectral) function, a Keldysh function is defined, but it is based on the symmetric commutator (denoted by {A,B}=AB+BA):

(23) $$iK_{AB}\left(x,p\right)\;=\;\frac{1}{2}\int \;dq\;e^{\frac{i}{\hbar }p\cdot q}\;\langle \left\{\hat{A}\left(x-q/2\right),\hat{B}\left(x+q/2\right)\right\}\rangle .$$

As a consequence, the Keldysh function properties by inverting the energy and momentum in its argument are as follows:

(24) $$iK_{AB}^{*}\left(x,p\right)\;=\;-iK_{B^{†}A^{†}}\left(x,p\right),\;iK_{AB}\left(x,-p\right)\;=\;iK_{BA}\left(x,p\right).$$

The behavior of the statistical expectation values of the number of particles, which is a particular case of using the creation and annihilation operators instead of *A* and *B*, follows some rules derived from the above. Considering bosons, for example, one uses Hermitean and scalar operators, $B=B^{†}=A=A†$, twice. We commonly denote them by Φ. In this case,

(25) $$S_{\Phi \Phi }^{*}\left(x,p\right)\;=\;S_{\Phi \Phi }\left(x,p\right),\;S_{\Phi \Phi }\left(x,-p\right)\;=\;-S_{\Phi \Phi }\left(x,p\right).$$

So, the spectral Wigner function is real and antisymmetric for the change in the sign of the four-momentum. This quantity counts negative energy states as minus.

To translate this result to the particle number (occupation number) quantities, we utilize the general relation between the commutator and anti-commutator. In the special case of $A=a^{†}$ and B=a fulfilling elementary commutation relations, we have for the bosons $2iK∼\left\{a^{†},a\right\}=2\hat{n}+1$ and $S∼[a^{†},a]=1$, while for the fermions, we have 2iK∼1 and $S∼2\hat{n}-1$. Based on this, we generalize the definition of occupation numbers by the relations

(26) $$iK_{AB}\left(x,p\right)\;=\;{\left(n_{AB}\left(x,p\right)\pm \frac{1}{2}\right)}^{\pm 1}S_{AB}\left(x,p\right).$$

with the plus sign for bosons and the minus sign for fermions. One obtains the sought relation between the quantum field occupations of negative and positive energy states based on this as follows:

(27) $$n_{AB}\left(x,-p\right)\;=\;\mp 1-n_{BA}\left(x,p\right).$$

This is the particle–hole symmetry for bosons (upper sign) and fermions (lower sign). Complex conjugation leads to another relation,

(28) $$n_{AB}^{*}\left(x,p\right)\;=\;n_{B^{†}A^{†}}\left(x,p\right).$$

This defined occupation number is real as long as ${\left(AB\right)}^{†}=B^{†}A^{†}=AB$, i.e., the operator product AB is Hermitean. For the traditional quantum counting operator, $A=a^{†}$, B=a, this is the case.

When the two operators coincide, A=B, then the particle–hole symmetry is expressed by containing the same quantity on the left and right hand side of the equation:

(29) $$n_{AA}\left(x,-p\right)\;=\;\mp 1-n_{AA}\left(x,p\right).$$

The antiparticle numbers are defined accordingly as

(30) $${\bar{n}}_{AB}\left(x,p\right)\;=\;\mp n_{BA}\left(x,-p\right),$$

in order to interpret the negative energy states.

Now comes the statistical part: we associate an exponential, eventually a generalized exponential function, to the ratio of particle and hole (antiparticle) states. The argument of the generalized exponential in a kinetic approach is usually the $\beta \cdot p=\beta u_{\mu }p^{\mu }$ Minkowski product for relativistic systems. The Jüttner distribution is generalized then by the ratio of our generalized occupation numbers:

(31) $$\frac{n_{AB}\left(x,p\right)}{{\bar{n}}_{AB}\left(x,p\right)}\;=\;\frac{n_{AB}\left(x,p\right)}{1\pm n_{AB}\left(x,p\right)}\;=\;e_{k}\left(-\beta \cdot p\right).$$

On the other hand, applying the same relation to a negative energy and opposite momentum state, the above formula by replacing $p^{\mu }$ with $-p^{\mu }$ reads as

(32) $$\frac{n_{AB}\left(x,-p\right)}{{\bar{n}}_{AB}\left(x,-p\right)}\;=\;\frac{1\pm n_{BA}\left(x,p\right)}{n_{BA}\left(x,p\right)}\;=\;e_{k}\left(+\beta \cdot p\right).$$

For the case A=B, self-correlation of an operator, this is only possible if

(33) $$e_{k}\left(-\beta \cdot p\right)\;e_{k}\left(\beta \cdot p\right)\;=\;1.$$

From Equation (31), it follows a given generalization of the Bose and Fermi distributions:

(34) $$n_{AB}\left(x,p\right)\;=\;\frac{e_{k}\left(-\beta \cdot p\right)}{1\mp e_{k}\left(-\beta \cdot p\right)}.$$

From its energy-momentum mirrored version, Equation (32), it follows another:

(35) $$n_{BA}\left(x,p\right)\;=\;\frac{1}{e_{k}\left(\beta \cdot p\right)\mp 1}.$$

Again, these definitions coincide only if the deformed exponential, which is used to replace the original exponential function, fulfills the special product rule Equation (33).

This result underlines the fact that the particle–hole (in the vacuum particle–antiparticle) symmetry applies not only to the Boltzmannian statistics but is also a basic requirement for the generalized occupation number functions of energy, describing the statistics of elementary particles or other types of quantum excitations.

In conclusion, we selected a very particular property of Kaniadakis’ generalized exponential function, namely its reciprocial property upon reflection of its argument, Equation (33), and emphasized its relation to the particle–hole symmetry, known in quantum field theory and reflected in the KMS relation. We also presented a generalization of this function class maintaining this special property and related it to another construction based on the Tsallis type generalization of the exponential function. By doing so, a slight generalization of the phase space occupation number density statistics revealed that more general correlation functions also satisfy a KMS-type relation, when taking into account the change in the order of non-identical operators.

## Data Availability

Not applicable.

## Abbreviations

The following abbreviations are used in this manuscript:

MDPI	Multidisciplinary Digital Publishing Institute
KMS	Kubo–Martin–Schwinger
